# High incidence of Y‐chromosome mosaicism in male and female individuals with mild malformation of cortical development with oligodendroglial hyperplasia in epilepsy

**DOI:** 10.1002/epi.70317

**Published:** 2026-05-30

**Authors:** Erica Cecchini, Till Hartlieb, Ahmed Gaballa, Katja Kobow, Mitali Katoch, Paraskevi Chasani, Georgia Vasileiou, Wiebke Hofer, Lea M. Reisch, Manfred Kudernatsch, Christian G. Bien, Roland Coras, Ingmar Blümcke, Lucas Hoffmann

**Affiliations:** ^1^ Department of Neuropathology University Hospital Erlangen, Friedrich‐Alexander‐Universität Erlangen‐Nürnberg, and partner of the European Reference Network EpiCARE Erlangen Germany; ^2^ Center for Pediatric Neurology, Neurorehabilitation, and Epileptology Schoen‐Clinic Vogtareuth Germany; ^3^ Research Program for Rehabilitation, Transition, and Palliation Paracelsus Medical University Salzburg Austria; ^4^ Department of Epileptology, Krankenhaus Mara, Bethel Epilepsy Center, Medical School Ostwestfalen‐Lippe (OWL) Bielefeld University Bielefeld Germany; ^5^ Institute of Human Genetics Friedrich‐Alexander‐Universität Erlangen‐Nürnberg Erlangen Germany; ^6^ Department of Human Genetics Hannover Medical School Hannover Germany

**Keywords:** brain somatic, epilepsy, oligodendroglia, *SLC35A2*, Y‐chromosome

## Abstract

**Objective:**

Mild malformation of cortical development with oligodendroglial hyperplasia in epilepsy (MOGHE) is an underrecognized pediatric cortical lesion associated with somatic X‐linked *SLC35A2* variants in approximately 50% of individuals. The genetic etiology in individuals without detectable *SLC35A2* mutations remains undefined, which limits our understanding of its pathomechanism and clinical characterization. The aim is to identify genetic correlates of MOGHE in individuals lacking pathogenic *SLC35A2* variants and to investigate the clinicopathological and genomic correlations.

**Methods:**

We investigated archival brain tissue of 29 individuals (19 males, 10 females) with histopathologically confirmed MOGHE. Twenty individuals carried *SLC35A2* variants, and nine individuals lacked detectable mutations in *SLC35A2* or other epilepsy‐related genes. Brain tissue samples were analyzed using DNA methylation‐derived copy number variation (CNV) analysis, polymerase chain reaction (PCR), and chromogenic/fluorescent in situ hybridization (ISH).

**Results:**

CNV analysis identified Y‐chromosomal material in five of 10 females (50%) and mosaic Y‐chromosome gains in 16 of 19 males (84%), confirmed by PCR and ISH, and associated with lesional tissue. Exploratory stratification of the cohort by *SLC35A2* status and Y‐chromosome gain revealed a tendency toward earlier seizure onset, multilobar involvement, and cognitive impairment in patients carrying both *SLC35A2* mutations and Y‐chromosome gain; however, these differences did not reach statistical significance in our small cohort.

**Significance:**

The combined presence of Y‐chromosomal material in females, mosaic Y‐chromosome gains in males, and *SLC35A2* variants represents a recurrent genomic feature in patients with MOGHE. These findings suggest that sex‐chromosome anomalies may contribute to the pathobiology of this epilepsy‐associated cortical malformation, highlighting the need to clarify the underlying molecular mechanisms of this clinicohistopathologic entity. Although the root cause, biological consequence, and clinical significance of brain somatic Y‐chromosomal gain remain to be further clarified, our findings identify a previously unrecognized genomic feature of MOGHE and provide a rationale for future research in MOGHE cohorts.

**Key points**

- In 29 individuals with MOGHE, brain somatic mosaic Y‐chromosome gains were identified in 84% of males and in 50% of females.
- Cohort stratification by Y‐chromosome gain and the *SLC35A2* status outlined three molecular subgroups in MOGHE.
- Individuals with both *SLC35A2* variants and Y‐chromosomal gain showed a more severe, although not statistically significantly, clinical course.
- The diagnostic portfolio for MOGHE should include *SLC35A2* and Y‐chromosomal testing to refine lesion characterization in future clinical practice and research.

## 1 INTRODUCTION

Mild malformation of cortical development with oligodendroglial hyperplasia in epilepsy (MOGHE) is an underrecognized brain lesion associated with drug‐resistant epilepsy. The lesion is most commonly identified in young children and is typically localized to the frontal lobe, where surgical resection remains the primary treatment for seizure control.^1^, ^2^ First described by the Erlangen Neuropathology group in 2017,^3^ MOGHE was included in the International League Against Epilepsy (ILAE) Classification of Focal Cortical Dysplasia in 2022.^4^ Histopathologically, MOGHE is defined by focal areas of increased oligodendroglial cell density and heterotopic neurons within the white matter, often accompanied by hypomyelination^1^, ^5^, ^6^ corresponding to areas of oligodendroglial hyperplasia.^6^ Patients with MOGHE present with two major clinical phenotypes: early onset epileptic encephalopathy, characterized by epileptic spasms and significant cognitive impairment, and drug‐resistant focal epilepsy, with onset typically in adolescence or young adulthood and normal or borderline cognitive function.^1^ Magnetic resonance imaging (MRI) often reveals mild cortical thickening, cortical/subcortical hyperintense T2/fluid‐attenuated inversion recovery signal at the gray‐white matter boundary, that is, the power button sign,^7^ and blurring or reduced corticomedullary differentiation in older patients,^7^, ^8^ reflecting the underlying white matter abnormality.

Deep‐targeted sequencing of resected brain tissue from multicenter pediatric cohorts identified pathogenic variants in the X‐linked *SLC35A2* gene in approximately 50% of MOGHE individuals.^1^, ^5^, ^9^, ^10^ Both germline de novo heterozygous and hemizygous pathogenic variants in *SLC35A2*, the latter also in a mosaic state, have been associated with congenital disorder of glycosylation type IIm. The disorder manifests with infantile onset seizures, developmental delay, intellectual disability, skeletal anomalies, and brain malformations.^11^ *SLC35A2* encodes a UDP‐galactose transporter responsible for moving galactose from the cytosol into the Golgi apparatus lumen. This process is essential for the glycosylation of proteins and lipids. Multiple studies have demonstrated a consistent association between *SLC35A2* variants and MOGHE,^5^, ^6^ as well as with intractable neocortical epilepsy,^12^ supporting its role in disease pathogenesis. Recent evidence demonstrated a significant reduction of SLC35A2 protein in the brain white matter of patients with *SLC35A2* nonsense variants,^6^ suggesting that the variant type may affect therapeutic response. However, interindividual variability of *SLC35A2* variants may underlie different responses to D‐galactose supplementation seen in previous clinical studies, with some patients exhibiting clinical benefit, whereas others remain refractory.^13^ Despite these insights, approximately half of MOGHE individuals lack identifiable pathogenic variants, leaving their genetic etiology unexplained.^5^, ^9^, ^14^, ^15^ Although sex chromosome aneuploidies have been increasingly linked to neurodevelopmental disorders and neurological impairment,^16^, ^17^, ^18^, ^19^, ^20^, ^21^ their role in epilepsy remains unexplored. Seizures have been consistently reported in Turner syndrome (45,X),^22^, ^23^, ^24^, ^25^, ^26^ Klinefelter syndrome (47,XXY),^27^, ^28^, ^29^, ^30^ 47,XYY syndrome (47,XYY),^31^, ^32^, ^33^ and 47,XXX syndrome (47,XXX).^34^, ^35^ Case reports and small series describe epilepsy or electroencephalographic abnormalities in a subset of patients with 47,XYY syndrome,^33^, ^36^ and population‐based studies suggest increased neurological morbidity in this group of individuals.^37^, ^38^, ^39^, ^40^ Based on these published observations, structural abnormalities or Y‐chromosome mosaicism might contribute to epileptogenesis. To date, no studies have systematically examined Y‐chromosome mosaicism in MOGHE. Our research revealed recurrent Y‐chromosome gains in male and female individuals, which were limited to the lesional white matter and absent from the nearby nonlesional cortex, suggesting lesion‐associated mosaicism as a novel and additional pathogenic mechanism in MOGHE.

## 2 MATERIALS AND METHODS

### 2.1 Cohort selection

Brain tissue samples were obtained from the Epilepsy Center at the Erlangen University Hospital, the Bethel Epilepsy Center, and the Schoen Clinic Vogtareuth (all in Germany). Cases were retrospectively selected from the diagnostic archive of the Neuropathological Institute of the University Hospital Erlangen. Inclusion criteria required a histopathological diagnosis of MOGHE, availability of *SLC35A2* mutation status, and corresponding DNA methylation data analyzed with the Conumee plots.^41^ All cases were reviewed by two board‐certified neuropathologists (I.B. and R.C.) according to the updated diagnostic ILAE criteria.^4^ Histopathologic features included increased oligodendroglial cell densities in subcortical white matter, which can be clustered, focal myelin loss confirmed by Nissl–Luxol fast blue staining, and heterotopic neurons within the white matter. The cohort comprised 29 patients (19 males and 10 females) with histologically confirmed MOGHE. Clinical data, including sex, age at epilepsy onset, MRI findings, and surgical outcome, were collected from medical records (Table 1). Both formalin‐fixed paraffin‐embedded (FFPE) and fresh‐frozen (FF) tissue were available for histopathology and molecular analyses. Ethics approval was granted by the Medical Faculty of Friedrich Alexander University Erlangen–Nuremberg (#193_18B, 18‐192_1‐Bio) and the University of Münster (2015‐088‐f‐S), with written informed consent obtained from all participants or legal guardians.

**TABLE 1:** Summary of the clinical and molecular data of patients included in this study.

ID	Sex	Brain lobes^a^	*SLC35A2* variant (hg19) NM_005660.3 (cDNA change)	NP_005651.1 (amino acid change)	*SLC35A2* variant type	VAF	Y‐gain (CNV log_2_ ratio)	Outcome (Engel)	NPSY (pre → post)
Group 1: SLC+/Y−, *n* = 8									
1.	M	Fr‐R	c.533G>T	p.(Gly178Val)	Missense	26.9%	No	IA	Mild/moderate → mild/moderate
2.	M	Fb‐R	c.335_339dup	p.(Lys114ArgfsTer32)	Frameshift insertion	52%	No	IA	Borderline → average
3.	M	Fr‐R	c.206C>T	p.(Thr69Leu)	Missense	14%	No	IA	Severe → severe
4.	F	Fm‐R	c.935C>T	p.(Ser312Phe)	Missense	8%	No^a^	IA	Borderline → mild
5.	F	Fr‐L	c.905C>T	p.(Ser302Phe)	Missense	8%	No^a^	IVA	Severe → severe
6.	F	Fop‐R	c.640G>C	p.(Gly214Arg)	Missense	8%	No	IVB	No intellectual impairment
7.	F	Fl‐R	c.515 T>C	p.(Leu172Pro)	Missense	3%	No	IIIA	No intellectual impairment
8.	F	Fr Gy‐R	c.364_365insC c.366delinsTCTC	p.(Tyr122SerfsTer6) p.(Tyr122_Thr123insLeu)	Frameshift (truncating) in‐frame insertion	15%	No	IIIA	NA
Group 2: SLC+/Y+, *n* = 12									
9.	F	Hm‐L	c.359_360delTC	p.(Leu120HisfsTer7)	Frameshift (truncating)	30%	Yes^b^, ^c^	IVA	NA
10.	F	Hm‐L	c.603_606dupAGGC	p.(Leu203ArgfsTer20)	Frameshift (premature stop)	21%	Yes^b^, ^c^	IA	Severe → moderate
11.	F	Fr‐R	c.640G>C	p.(Gly214Arg)	Missense	n.a.	Yes (0,0)^c^	IA	NA
12.	F	Hm‐L	c.935C>T	p.(Ser312Phe)	Missense	33%	Yes (0,3)^c^	IA	NA
13.	M	Hm‐L	c.424C>T	p.(Gln142X)	Nonsense	19.4%	Yes (0,4)	IA	Severe → severe
14.	M	Hm‐R	c.569_572del	p.(Gly190AlafsTer158)	Frameshift (premature stop)	41%	Yes^d^	IIIA	Severe → severe
15.	M	TPO‐L	c.832C>T	p.(Gln278Ter)	Nonsense	48%	Yes (0,9)	IA	Severe → severe
16.	M	FT‐R	c.675dupA	p.(Gly226ArgfsTer29)	Frameshift (premature stop)	40%	Yes (0,9)	IA	Moderate → borderline
17.	M	Fr‐L	c.113delT	p.(Ile38AsnfsTer3)	Frameshift (early termination)	41%	Yes (0,9)	IA	Moderate → moderate
18.	M	FT‐L	c.580_616dup	p.(Val206AlafsTer28)	Frameshift (premature stop)	9%	Yes (1,0)	IIIA	NA
19.	M	Fr‐L	c.665_667del	p.(Lys222del)	In‐frame deletion	32.6%	Yes (0,8)	IIIA	NA
20.	M	Hm‐L	c.322C>T	p.(Gln108Ter)	Nonsense	25%	Yes (1,0)	IA	Severe → moderate
Group 3: SLC−/Y+, *n* = 9									
21.	F	Fr‐R	wt	—	No variant	—	Yes (1,0)^c^	NA	NA
22.	M	Fr‐L	wt	—	No variant	—	Yes (0,4)	IA	Severe → NA
23.	M	Fp/Fb‐L	wt	—	No variant	—	Yes (0,4)	IA	Moderate → moderate
24.	M	TPO‐L	wt	—	No variant	—	Yes (0,4)	IVA	Severe → severe
25.	M	Fb‐L	wt	—	No variant	—	Yes (0,8)	IA	No intellectual impairment
26.	M	T Gy‐R	wt	—	No variant	—	Yes (0,8)	NA	NA
27.	M	Fr‐R	wt	—	No variant	—	Yes (0,9)	IIA	Borderline → borderline
28.	M	Fr‐R	wt	—	No variant	—	Yes (1,0)	IA	Moderate → mild
29.	M	Hm‐L	wt	—	No variant	—	Yes (1,0)	IA	NA

Abbreviations: CNV, copy number variation; F, female; Fb, frontobasal; FISH, fluorescence in situ hybridization; Fl, frontolateral; Fm, frontomesial; Fop, fronto‐orbitopolar; Fp, frontopolar; Fr, frontal; FT, frontotemporal; Gy, gyrus; Hm, hemispheric; L, left; M, male; NPSY, neuropsychology (refers to the neuropsychological assessment before and after surgery); NA, not available; R, right; SLC+/Y−, *SLC35A2* variants without detectable Y‐chromosome gains; SLC+/Y+, *SLC35A2* variants in conjunction with Y‐chromosome gains; SLC−/Y+, lacking *SLC35A2* mutations but exhibiting Y‐chromosome gains; T, temporal; TPO, temporoparieto‐occipital; VAF, variant allele frequency; wt, wild type.

^a^ Refers to the affected brain lobes as described in the presurgical magnetic resonance imaging report.

^b^ The CNV profile for this patient was inconclusive and was subsequently validated by FISH and PCR.

^c^ Confirmed by PCR analysis.

^d^ The CNV profile for this patient was inconclusive and was subsequently validated by FISH.

### 2.2 Genetic background

All 29 cases had been reported in previous studies, with 20 (68.9%) carrying brain somatic pathogenic variants in the X‐linked *SLC35A2* gene.^1^, ^6^, ^9^, ^14^, ^42^ Prior deep‐targeted sequencing identified eight missense, eight frameshift, three nonsense, and two in‐frame variants, with variant allele frequencies from 3% to 52%; one patient carried two variants. The remaining nine individuals lacked pathogenic variants in *SLC35A2* or other epilepsy‐related genes. Matched blood samples were unavailable, although prior evidence supported a brain somatic origin for *SLC35A2* variants in MOGHE tissue. Fourteen individuals were analyzed using a targeted gene panel,^14^ and the remaining 15 underwent exome sequencing^9^ using the standard MoChA (Mosaic Chromosomal Alterations) pipeline.

### 2.3 Neuropsychological assessment

Neuropsychological evaluations were performed in 13 of 17 German‐speaking individuals at the Schoen Clinic Vogtareuth and in all individuals at the Bethel Epilepsy Center, following standardized longitudinal protocols. Assessments were conducted preoperatively and at 6 and 24 months after surgery using age‐appropriate instruments: the Hamburg‐Wechsler Intelligence Test for Children, Wechsler Intelligence Tests for Adults, Bayley Scales of Infant Development II, and Vineland Adaptive Behavior Scales. Cognitive outcomes were categorized according to International Classification of Diseases, 10th Revision criteria: no impairment/average (intelligence quotient [IQ] ≥ 85), borderline (IQ = 70–84), mild (IQ = 50–69), moderate (IQ = 35–49), or severe/profound (IQ < 35). At the Bethel Center, comparable instruments were used, including the Bayley Scales of Infant Development II, Wechsler Preschool and Primary Scale of Intelligence or Wechsler Intelligence Scale for Children, and McCarthy Scales of Children's Abilities. Adult patients were tested using the Vocabulary Test and Logical Reasoning Subtest to estimate general intellectual ability.

### 2.4 DNA methylation and copy number variation analysis

Genome‐wide DNA methylation profiling was performed on FF tissue using the Illumina Infinium MethylationEPIC BeadChip (850 K). Raw IDAT files were processed in R (v4.3) with the minfi package (v1.46.0) using Noob normalization and alignment to the hg19 reference genome. Copy number variation (CNV) profiles were generated using an optimized version of the conumee R package (github.com/FAU‐DLM/conumee.git) and normalized against in‐house reference controls. Gene annotations were based on EnsDb.Hsapiens.v86, restricted to protein‐coding genes. Probes were filtered for high‐confidence loci according to the Illumina EPIC annotation file (EPICanno.ilm10b4.hg19). Y‐chromosomal gains were defined based on log_2_ ratio thresholds of >.4 in males and >.0 in females. Quality control included probe coverage thresholds, exclusion of low‐confidence regions, and matched‐control normalization. Genome‐wide and chromosome‐specific CNV plots were generated for all samples.

To assess the specificity of Y‐chromosomal gains, an independent comparison cohort of 147 DNA methylation‐derived CNV profiles was included, comprising 33 mild malformations of cortical development (mMCD; 16 females, 17 males), 47 focal cortical dysplasia type II (FCDII; 18 females, 29 males), and 67 low‐grade epilepsy‐associated tumor (LEAT) samples (27 females, 40 males). The samples were derived from epilepsy surgery cases and were previously reported by Jabari et al. (Table 2).^41^ However, sex‐chromosome‐specific CNV analyses were not performed in this prior study.

**TABLE 2:** Independent cohorts of 147 epileptogenic brain lesions included in this study.

Diagnosis	*n*	F	M
mMCD	33	16	17
LEAT	67	27	30
FCDII	47	18	29
Total	147	61	76

Abbreviations: F, female; FCDII, focal cortical dysplasia type II; LEAT, Low‐grade epilepsy‐associated tumor; M, male; mMCD, mild malformation of cortical development; *n*, number of included samples.

### 2.5 Chromogenic and fluorescent in situ hybridization

Chromogenic in situ hybridization (CISH) was performed using ZytoDot 2C CEN X/Y probes (ZytoVision), with the X probe targeting Xp11.1–q11.1 (red signal) and the Y probe targeting Yp11.1‐q11.1 (green signal). Signal detection used anti‐DIG/DNP antibody mixes with HRP/AP polymer systems and chromogenic substrates. Fluorescence in situ hybridization (FISH) employed the ZytoLight CEN X/Y dual‐color probe, where ZyGreen labeled the X centromere, and ZyOrange labeled the Y centromere. One male and one female temporal lobe epilepsy case served as non‐MOGHE controls. Nuclei were counterstained with hematoxylin, and slides were digitized using a Hamamatsu S60 scanner. FISH imaging was performed on an Olympus BX51 fluorescence microscope with a ColorView 2 camera.

### 2.6 Polymerase chain reaction amplification of Y‐specific sequences

The presence of Y‐chromosomal material was validated by polymerase chain reaction (PCR) amplification of the sex‐determining region Y (SRY) locus. Manual microdissection of lesional white matter and adjacent nonlesional cortex from the same FFPE block allowed intrasample comparison. DNA extraction was performed using the QIAamp DNA Mini Kit (Qiagen). A two‐step PCR was used to enhance sensitivity in low‐yield samples. Primers amplified a 266‐bp SRY fragment (forward: 5′‐GGA AGC AAA CTG CAA TTC TTC GG‐3′; reverse: 5′‐GAT AGA GTT GAA GCG ACC CAT GAA‐3′). Products were visualized on 3% agarose gels with RedSafe nucleic acid stain under ultraviolet illumination. Each run included male and female controls with and without MOGHE. Qualitative PCR validation of Y‐positive female cases was conducted in seven patients. The whole procedure was performed by a female operator to avoid any possible contamination with Y genetic material, and all runs were performed in three technical replicates.

### 2.7 Statistical analysis

Statistical analyses were performed using GraphPad Prism (v10.1) and R (v4.3). Continuous variables were summarized as medians and interquartile ranges (IQRs), and categorical variables as counts and percentages. Group comparisons of nonparametric variables, including age at seizure and lesion size, were assessed using the Kruskal–Wallis test. Associations between categorical variables were examined using Fisher tests. A two‐tailed *p*‐value < .05 was considered statistically significant. Because of the limited sample size, the associations of the clinical data (lesion size, age at epilepsy onset, and neuropsychological assessment) did not reach statistical significance, and these results indicate rather a tendency.

## 3 RESULTS

### 3.1 DNA methylation‐derived CNV analysis reveals Y‐chromosomal gains

**FIGURE 1:**
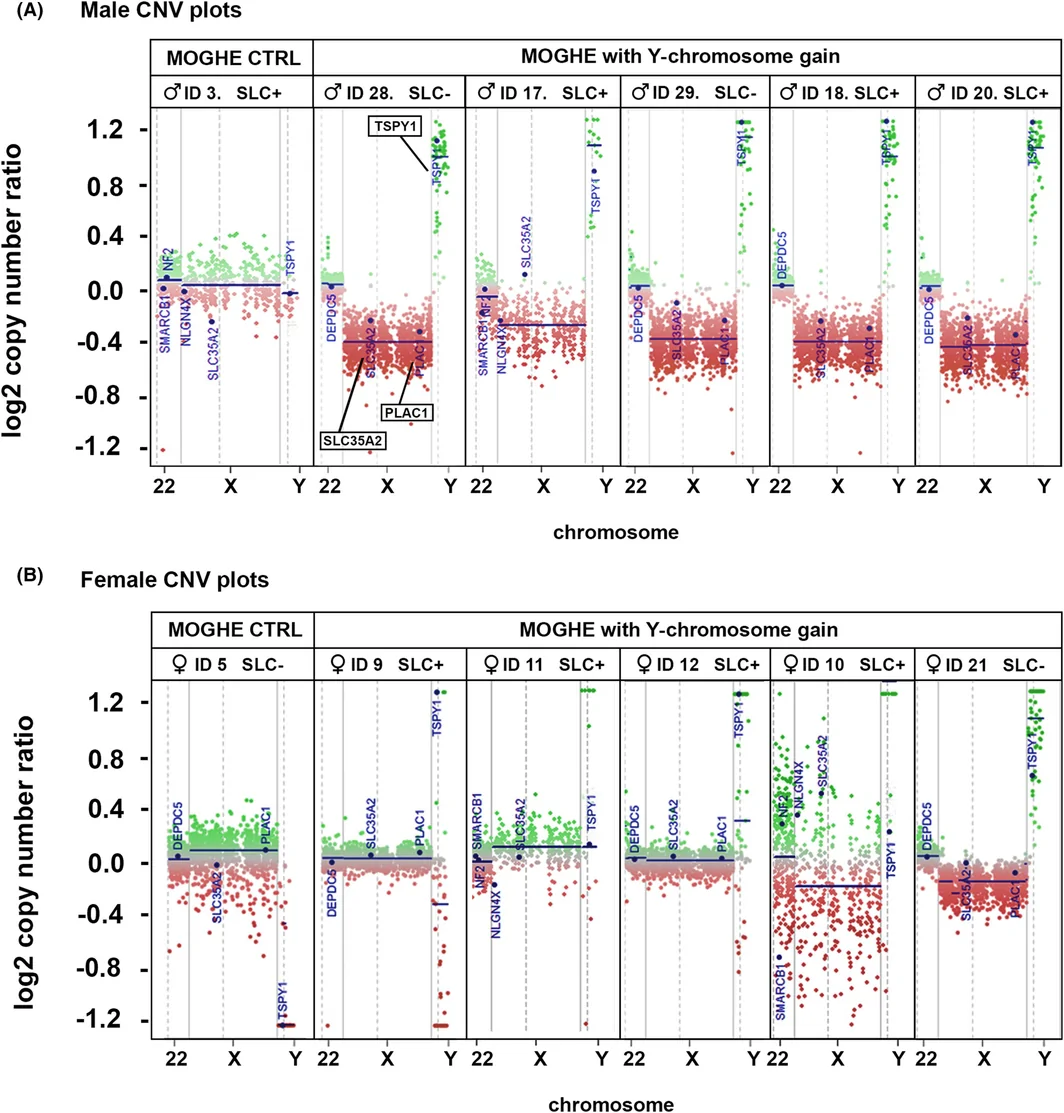
Copy number variation (CNV) plots of males and females with mild malformation of cortical development with oligodendroglial hyperplasia in epilepsy (MOGHE) and Y‐chromosome gain. For both panels, the x‐axis indicates chromosomes 22, X, and Y, providing spatial reference within the CNV plots. The y‐axis represents log_2_ copy number ratios, with the baseline corresponding to the blue line at log_2_ = .0. Annotations denote the mutational status of *SLC35A2*: “SLC+” indicates a pathogenic variant; “SLC−” indicates wild type. Gene names are displayed in blue, according to the standard Conumee CNV plot format. (A) CNV profiles of six males with MOGHE (IDs 3, 28, 17, 29, 18, 20). The first case shows a normal CNV pattern (Y‐chromosome at log_2_ ratio = .0), whereas the remaining five cases exhibit a gain of the Y‐chromosome, consistently detected between .8 and 1.0 on the log_2_ copy number ratio scale. (B) CNV profiles of six females with MOGHE (IDs 5, 9, 11, 12, 10, 21). The first case shows no Y‐chromosome gain, whereas the remaining five cases demonstrate Y‐chromosome gains ranging from .0 to 1.0 on the log_2_ copy number ratio scale. ID 9 exhibits an intermediate CNV profile, and ID 10 displays low‐quality CNV data, necessitating validation by complementary methods (fluorescence in situ hybridization, polymerase chain reaction). Magnified inset windows highlight representative loci, highlighting *TSPY1* (Y‐chromosome) and *PLAC1/SLC35A2* (X chromosome). These genes consistently occupy the same genomic positions across all CNV plots. Insets are provided once as representative examples to avoid visual overcrowding of the figure. CTRL, control.

CNV analysis based on DNA methylation profiles was performed on 29 individuals with MOGHE (19 males, 10 females). Significant gains of Y‐chromosome sequences were detected in 16 of 19 males (84.2%) with log_2_ ratios from +.4 to +1.2, exceeding thresholds for CNV calling (Figure 1A). Unexpectedly, Y‐chromosomal gains were detected in five of 10 females (50%), with log_2_ ratios from +.0 to +1.0 (Figure 1B). In two females (ID 9 and 10) and one male (ID 14), CNV profiles were inconclusive and confirmed by FISH and/or PCR. No significant CNVs were detected on autosomes or the X chromosome. Individual CNV plots for all individuals are shown in Figure S1. Within the independent comparison cohort of 147 DNA methylation‐derived CNV profiles from other epileptogenic brain lesions (Table 2), Y‐chromosomal gains were identified in eight cases (5.4%), and exclusively in males. These included one of 33 mMCD samples, two of 47 FCDII samples, and five of 67 LEAT samples. Pairwise comparisons using Fisher exact test demonstrated a significant enrichment of Y‐chromosomal gain in MOGHE compared with mMCD (odds ratio [OR] = 13.4, Bonferroni‐adjusted *p*‐value [adj *p*‐value] = .007), FCDII (OR = 9.4, adj *p*‐value = .003), and LEAT (OR = 5.2, adj *p*‐value = .009).

Based on the integrated analysis of *SLC35A2* mutational status and Y‐chromosome CNVs, the cohort was stratified into three distinct subgroups: Group 1, individuals harboring pathogenic *SLC35A2* variants without detectable Y‐chromosome gains (*n* = 8); Group 2, individuals carrying pathogenic *SLC35A2* variants in conjunction with Y‐chromosome gains (*n* = 12); and Group 3, individuals lacking *SLC35A2* mutations but exhibiting Y‐chromosome gains (*n* = 9; Table 1).

### 3.2 Clinical findings in genetically defined MOGHE subgroups

Median age at seizure onset differed across the three molecularly defined subgroups, although these comparisons were exploratory and did not reach statistical significance (Kruskal–Wallis test, *H* = 2.786, *p* = .25). Patients with Y‐chromosome gains tended to present with an earlier disease onset (Kruskal–Wallis test, *H* = 2.786, *p* = .25). Median onset ages were 3.9 years (IQR = .54–13.5, range = .16–24 years) for Group 1, .58 years (IQR = .455–1.04, range = .25–3 years) for Group 2, and 2.0 years (IQR = .54–5.25, range = .33–12 years) for Group 3.

**FIGURE 2:**
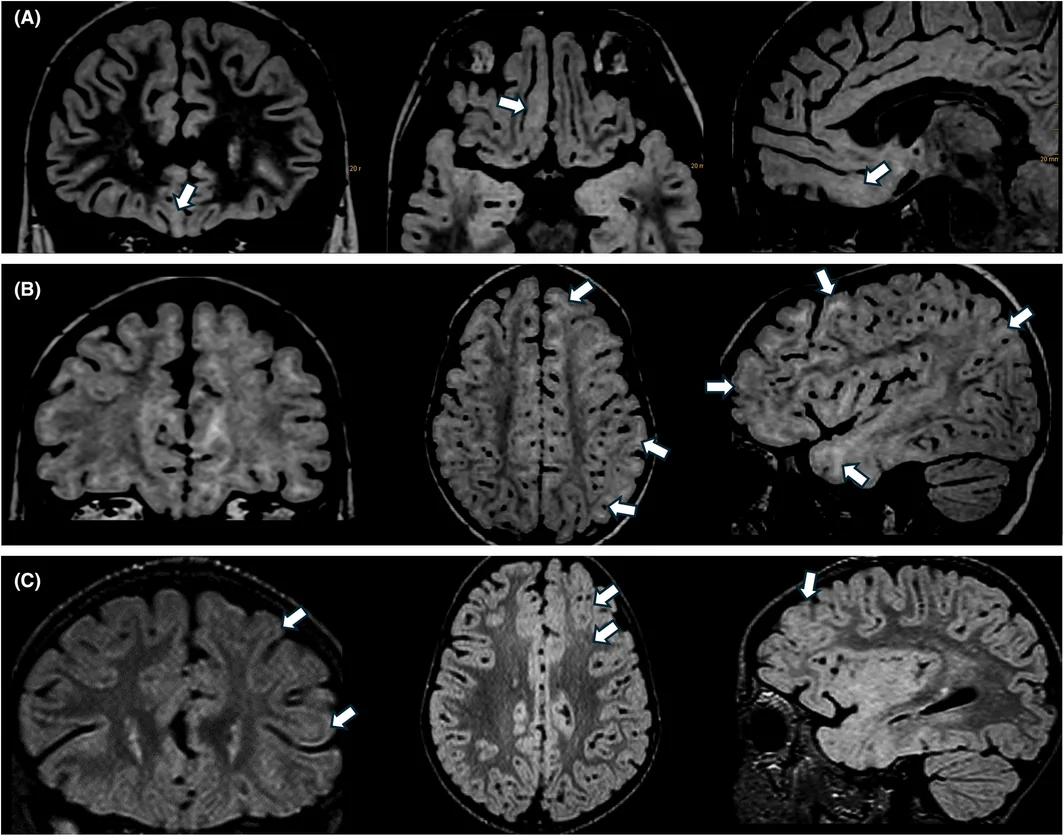
Representative magnetic resonance imaging (MRI) of individuals with mild malformation of cortical development with oligodendroglial hyperplasia in epilepsy. Representative MRI in coronal, axial, and sagittal planes demonstrates lesion extent and distribution. White arrows mark the lesions. (A) Circumscribed right orbitofrontal lesion. (B) Extensive left hemispheric lesion with an enhanced power button sign. (C) Left frontal lesion.

Lesion size, according to the presurgical MRI, varied among groups. Group 1 typically exhibited small, well‐circumscribed frontal lesions, often frontobasal, frontomesial, or orbitopolar (Figure 2A). Group 2 exhibited larger lesions, with six of 12 hemispheric and three multilobar (Figure 2B). Group 3 had intermediate‐to‐large lesions, including one hemispheric case, and the majority involved the entire frontal lobe (Figure 2C). Larger lesions tended to correlate with earlier seizure onset, especially in Group 2.

Neuropsychological assessments were available for 20 patients (Table 1), who did not show statistically significant differences between the molecularly defined MOGHE subgroups. In Group 1, however, intellectual function ranged from severe impairment to average, with most patients remaining stable or improved slightly postoperatively (e.g., borderline → average). Two patients showed no impairment before or after surgery. Group 2 exhibited more pronounced deficits, primarily moderate to severe, with three improving. Group 3 showed heterogeneous outcomes, from severe disability to normal cognition, with mostly stable trajectories and occasional improvement. Overall, postoperative cognition remained stable or modestly improved, with Group 1 showing the best cognitive outcomes, Group 2 the most severe impairment, and Group 3 the most variable.

Histopathology revealed no significant differences between MOGHE subgroups. Mean oligodendroglial cell densities (OLIG2‐positive nuclei/mm^2^) were similar (Group 1: 2416; Group 2: 2376; Group 3: 2153; *p* > .05). Proliferative activity was uniformly low (Ki‐67 < 1% in all samples). Hypomyelination on Nissl–Luxol Fast Blue staining was least prominent in Group 1, present in seven of 12 in Group 2 cases, and most frequent in Group 3, consistent with prior findings that hypomyelination decreases with patient age.^6^

### 3.3 In situ hybridization confirms Y‐chromosome gains in lesional tissue

**FIGURE 3:**
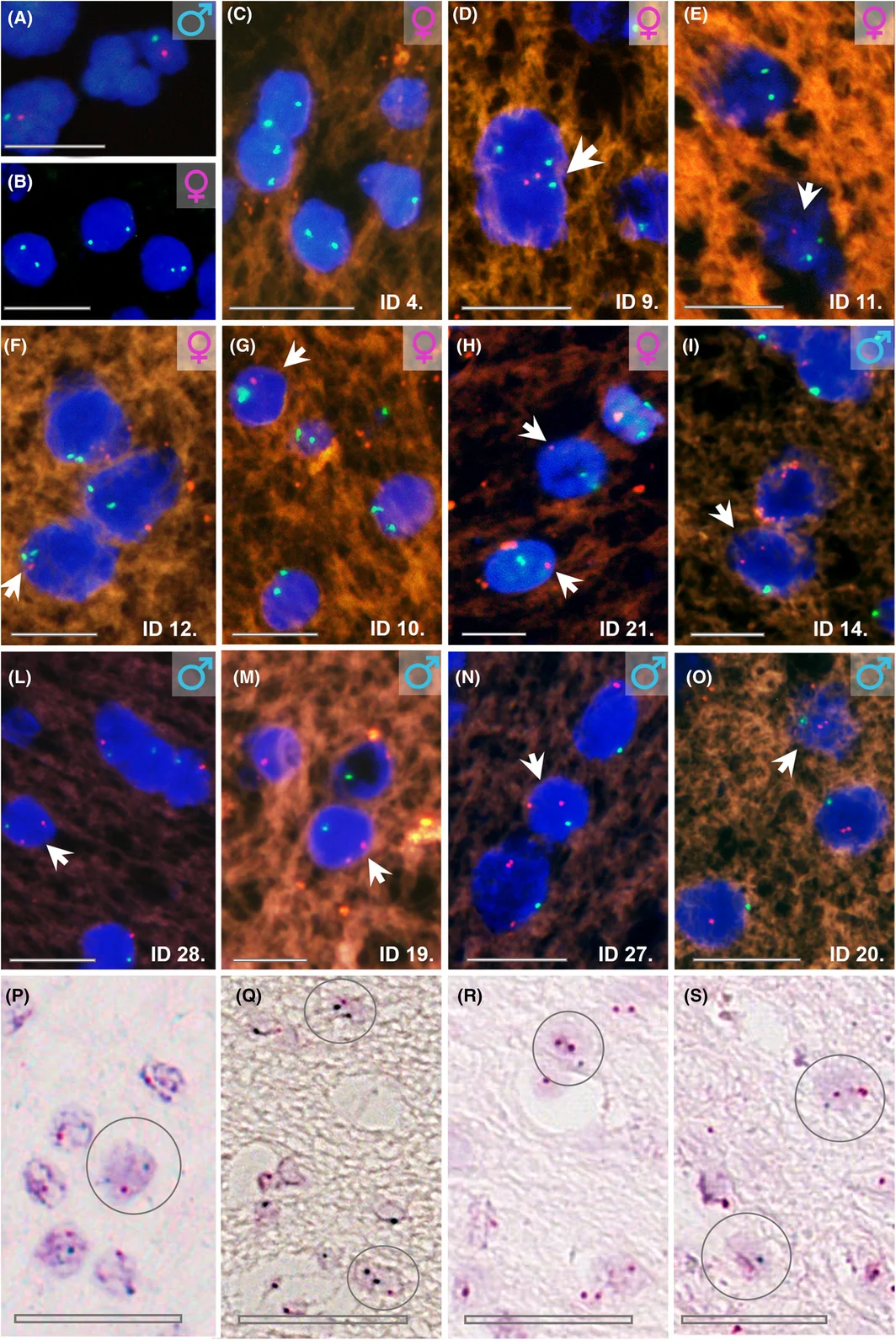
Fluorescence in situ hybridization (FISH)/chromogenic in situ hybridization (CISH) for X and Y probes in control and brain tissue samples with mild malformation of cortical development with oligodendroglial hyperplasia in epilepsy (MOGHE). Dual‐color fluorescence (FISH) and chromogenic (CISH) in situ hybridization using centromeric X and Y probes demonstrates Y‐chromosome gains in MOGHE. In FISH (A–O), the X chromosome = green, the Y‐chromosome = red. (A, B) Control brain tissue: male (XY) with one X and one Y signal; female (XX) with two X signals and no Y signal. (C) Female MOGHE (ID 4) with copy number variation plot negative for Y‐chromosomal gain, showing two X signals. (D–H) Female MOGHE cases (IDs 9, 11, 12, 10, 21) showing two X and one Y signal in lesional nuclei (white arrowheads). (I–O) Male MOGHE cases (IDs 14, 28, 19, 27, 20) with one X and two Y signals (white arrowheads). (P–S) CISH validation of lesional white matter shows consistent patterns of Y‐chromosome gain (gray circles); probe colors are reversed (X = red, Y = green). Scale bars: 5–10 μm (A–O); 25 μm (P–S).

FISH and CISH were performed using dual‐color centromeric X/Y probes (ZytoVision) to validate CNV findings at the cellular level. FISH visualized Y‐positive nuclei predominantly in regions of oligodendroglial hyperplasia (Figure 3A), with X signals in green and Y signals in red. In female individuals with CNV‐detected Y gains, including one with intermediate CNV results (ID 9), FISH confirmed Y‐positive nuclei in all cases, verifying Y‐chromosomal sequences even when CNV plots were inconclusive. Scattered nuclei within lesional white matter displayed an additional Y signal in otherwise XX cells. Female CNV‐negative cases showed only two X signals, serving as internal controls. Male individuals with Y‐chromosome gains nuclei displayed one X and two Y signals, confirming extra Y material. CISH provided orthogonal validation, supporting the FISH findings, although fluorescence imaging offered higher resolution (Figure 3B). Due to frequent nuclear truncation in FFPE sections, quantitative counts were not feasible and remained qualitative. Importantly, Y‐positive nuclei were associated with lesional white matter and absent in overlying cortex or CNV‐negative controls, supporting spatial confinement to oligodendroglial hyperplasia. To preliminarily assess the structural integrity of the Y‐chromosome, we performed additional FISH analyses in five females and six males using probes targeting CENX, CENY, and Yq12 (DYZ1). In all females (*n* = 5), colocalization of CENY and Yq12 signals was observed, consistent with a gain of an intact Y‐chromosome. Similarly, in 50% of males (*n* = 3), colocalization supported the presence of an intact additional Y‐chromosome.

In the remaining males (*n* = 3), amplification of Yq12 signals and partial spatial separation from CENY was detected, suggesting either long‐arm amplification of a Y‐chromosome or integration of Yq12 material into other chromosomal regions (Figure 2). However, the partial spatial separation of two signals, particularly when minimal, in interphase FISH analyses may reflect variations in chromatin configuration or nuclear architecture instead of a structural chromosomal abnormality. Therefore, these observations should be considered preliminary and interpreted with caution.

### 3.4 PCR validation of Y‐chromosome sequences in female patients with MOGHE

**FIGURE 4:**
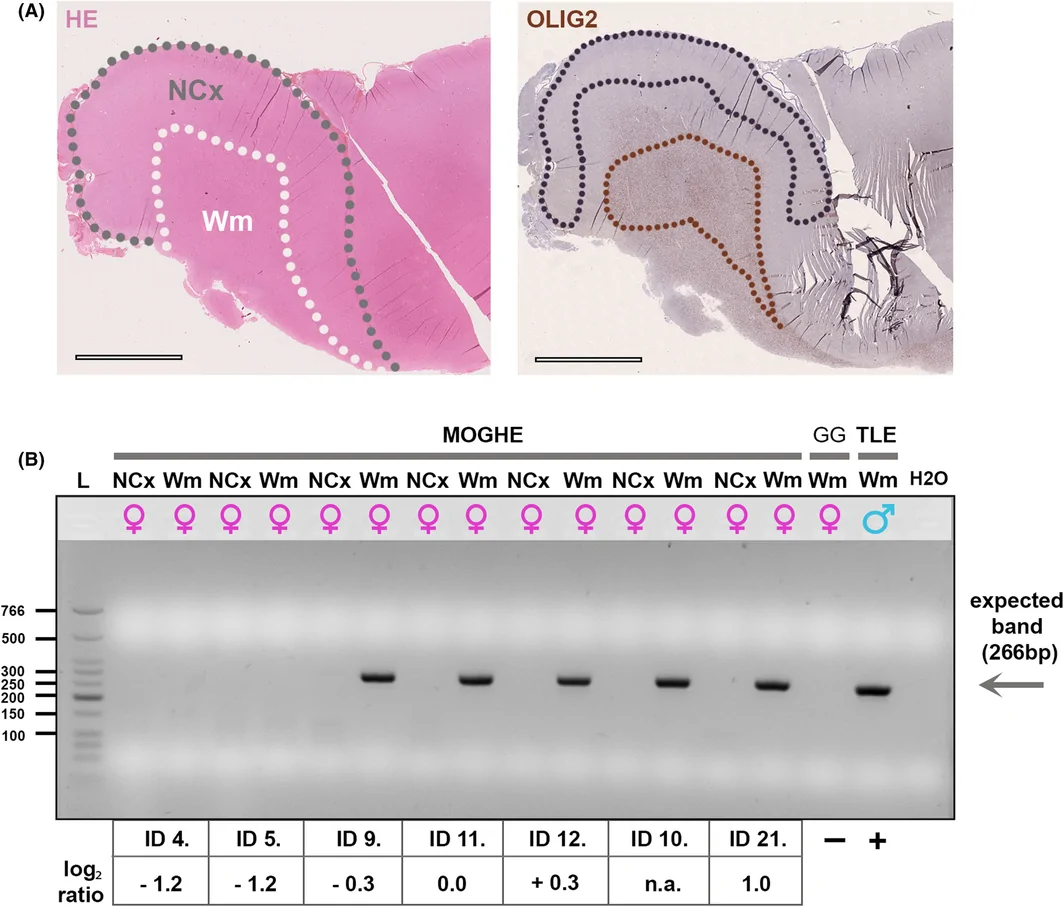
Polymerase chain reaction (PCR) amplification of the *SRY* locus in females with mild malformation of cortical development with oligodendroglial hyperplasia in epilepsy (MOGHE) and controls. (A) Histological regions selected for microdissection before PCR. Hematoxylin and eosin (HE) staining distinguishes cortex (NCx) and white matter (Wm); OLIG2 immunostaining highlights oligodendroglial hyperplasia. (B) PCR amplification of a 266 bp SRY fragment detected in lesional white matter but not cortex, suggesting lesion‐associated Y‐chromosomal sequences. All copy number variation (CNV)‐positive and intermediate female cases were *SRY*‐positive; CNV‐negative and ganglioglioma (GG) controls were negative. Male temporal lobe epilepsy (TLE) served as a positive control. Scale bars: 5 mm (A); DNA ladder sizes (L) are indicated in panel B.

PCR amplification of the SRY locus was performed on lesional and nonlesional DNA from seven female individuals (Table 1). The subset included four females with CNV‐confirmed Y gains, one with intermediate CNV results, and two CNV‐negative cases (IDs 4 and 5) as controls. Additional negative DNA control was from a female with ganglioglioma, and positive DNA control from a male with temporal lobe epilepsy (Figure 4). Manual microdissection of white matter and cortex was guided by histology and OLIG2 immunostaining (Figure 4A). Y‐specific amplicons were detected in all CNV‐positive and intermediate CNV females, confirming Y sequences even when CNV data were ambiguous. No amplification occurred in CNV‐negative MOGHE cases or in negative controls. Within cases, Y‐specific amplicons were consistently detected in lesional white matter but absent in paired cortex, supporting lesion‐associated localization within the brain specimen and providing an intrasample control in the absence of peripheral tissue (Figure 4B).

The presence of SRY‐specific amplicons in lesional but not nonlesional tissue corroborated CNV and in situ hybridization (ISH) results, indicating lesion‐associated Y‐chromosome gain mosaicism. No Y signal was detected in CNV‐negative cases by ISH or PCR. Collectively, CNV, FISH/CISH, and PCR analyses confirmed lesion‐associated Y‐chromosome gains in male and female individuals with MOGHE, localized to oligodendroglial hyperplasia.

## 4 DISCUSSION

Recurrent, lesion‐associated gains of Y‐chromosomal sequences were identified in 72% of surgically resected brain tissues from 29 individuals with histopathologically confirmed MOGHE, detected in 84% of males and 50% of females. Using three complementary approaches, Y‐chromosome gains were consistently observed in lesional white matter and enriched in oligodendroglial cell clusters. Together with X‐linked *SLC35A2* variants, these findings identify a shared genomic feature across the MOGHE cohort (*SLC35A2* variants alone [28%], Y‐chromosome gain alone [31%], or both [41%]), without implying a definitive pathogenic role. Analysis of an independent comparison cohort comprising 147 epileptogenic brain lesions demonstrated that Y‐chromosomal gains were significantly enriched in MOGHE, but rare in mMCD, FCDII, and LEAT samples.

MOGHE is a recently defined developmental lesion primarily associated with brain somatic *SLC35A2* variants, which account for approximately half of reported cases.^1^, ^5^, ^9^, ^14^, ^43^ The present findings expand this molecular spectrum by identifying Y‐chromosome gain mosaicism as an additional, recurrent genomic observation. Whereas somatic Y‐chromosome loss in blood and brain has been reported as an age‐related event,^44^ focal gain of Y material within cortical malformations has not been described so far, and its biological significance remains uncertain. The co‐occurrence of *SLC35A2* variants and Y‐chromosome gains in nearly half of the individuals suggests convergent mechanisms affecting glycosylation, oligodendroglial proliferation, or cortical circuit organization. These data also support the broader concept that sex‐chromosome alterations, beyond classical aneuploidies such as 47, XXY or Klinefelter syndrome, can contribute to epileptogenesis.

Although all samples with MOGHE exhibited consistent histopathologic features, clinically defined subgroups showed different tendencies in disease onset and lesion extent. The subgroup carrying both *SLC35A2* variants and Y‐chromosome gains represented the largest proportion of cases (41% of the cohort) and showed a tendency toward earlier median seizure onset, larger lesions, and more pronounced cognitive impairment, without reaching statistical significance. Patients with Y‐chromosome gains alone also tended to present earlier and with larger lesions than those carrying *SLC35A2* variants alone. However, these observations describe a tendency that did not reach statistical significance in this small cohort and will need confirmation in independent large studies.

The presence of Y‐chromosomal material in females with MOGHE raises questions regarding its biological origin and whether it reflects brain somatic mosaicism or microchimerism. Early embryonic sex‐chromosome mosaicism with subsequent loss of Y in most cells represents a possible explanation. A speculative possibility is residual microchimerism from a vanished male co‐twin, which could theoretically result in persistent Y‐positive cell populations in selected tissues; however, this scenario cannot be assessed in the absence of clinical information on twin gestation or analysis of peripheral tissues. Y‐positive cell populations have also been described in phenotypic females in the context of sex‐chromosome mosaicism^24^, ^25^, ^45^ and in maternal brain tissue due to male microchimerism, possibly arising from fetal–maternal cell exchange.^46^, ^47^ In males, Y‐chromosome gains are likely the result of mitotic nondisjunction during early neurodevelopment,^48^ producing mosaic populations of Y‐positive oligodendroglia. The confinement of Y‐positive nuclei to lesional white matter, observed in this study, may reflect either clonal expansion within a permissive microenvironment or selective survival of Y‐positive cells,^49^ but alternative explanations, including epiphenomenal accumulation within abnormal tissue, cannot be excluded. The functional consequences of the detected Y‐chromosomal material remain unresolved, therefore, and any potential influence on oligodendroglial biology or epileptogenesis cannot be demonstrated by the present data. These results extend prior evidence, however, linking sex chromosome abnormalities to neurodevelopmental disorders and epilepsy^16^, ^33^, ^37^, ^40^ and identifying mosaic Y‐chromosome gain as a new feature within lesional epilepsy.

Our findings uncover a novel genomic feature in MOGHE, highlighting a potential role for sex‐chromosome anomalies in cortical malformations and providing a framework for future investigations in larger cohorts to clarify their clinical and pathogenic relevance. They also emphasize the need for molecular analyses in the diagnostic workup of drug‐resistant pediatric epilepsy,^4^ and a better definition of the molecular basis of MOGHE will open new avenues for future research into targeted therapeutic strategies for individuals with *SLC35A2*‐related glycosylation defects or chromosomal mosaicism‐associated cortical malformations.

### 4.1 Limitations

This study has several limitations. The cellular origin of Y‐positive nuclei could not be determined, leaving unresolved whether these cells represent brain‐resident mosaicism or microchimeric infiltrates. The analysis was restricted to brain tissue, precluding assessment of systemic distribution of Y‐positive cells. This limitation reflects the retrospective nature of the cohort, which spans surgeries performed before the establishment of a long‐term storage of peripheral tissue routine (e.g., mucosal swaps and blood). The modest cohort size (*n* = 29) limited statistical power to detect significant genotype–phenotype associations, for example, with respect to disease onset, lesion size, and cognitive impairment. Furthermore, the functional significance of Y‐chromosome gains, whether causal in lesion formation or a secondary phenomenon, remains uncertain. Future studies employing single‐cell genomic and transcriptomic analyses are required to clarify the structural nature, lineage origin, and biological impact of Y‐chromosome mosaicism in MOGHE.

## 5 CONCLUSIONS

Y‐chromosome gains represent a recurrent, lesion‐associated genomic finding in MOGHE in both males and females and, based on the currently available data, a brain somatic event confined to regions of oligodendroglial hyperplasia. Whether these gains contribute to lesion development or represent secondary changes within abnormal tissue remains unresolved. Together with *SLC35A2* variants, these alterations provide a shared genomic correlate in all individuals with MOGHE. The findings broaden our current understanding of the genetic mechanisms underlying cortical malformations and support a role for sex‐chromosome biology in the pathogenesis of lesional epilepsy.

## AUTHOR CONTRIBUTIONS

**Erica Cecchini**: Investigation; visualization; conceptualization; writing—original draft preparation. **Till Hartlieb**: Resources, investigation. **Ahmed Gaballa**: Resources, investigation. **Katja Kobow**: Data curation, investigation. **Mitali Katoch**: Formal analysis. **Paraskevi Chasani**: Formal analysis. **Georgia Vasileiou**: Conceptualization. **Wiebke Hofer**: Resources, investigation. **Lea M. Reisch**: Resources, investigation. **Manfred Kudernatsch**: Resources. **Christian G. Bien**: Resources. **Roland Coras, Ingmar Blümcke**: Conceptualization; writing—review and editing; supervision. **Lucas Hoffmann**: Conceptualization; writing—review and editing; supervision. All authors have reviewed the manuscript.

## FUNDING INFORMATION

I.B., K.K., L.H., and E.C. were supported by the Deutsche Forschungsgemeinschaft (German Research Foundation; project number 460333672–CRC1540 Exploring Brain Mechanics). Mi.K. was supported by grants of the Else Kröner‐Fresenius‐Stiftung (project number 2021_EKeA.33).

## CONFLICT OF INTEREST STATEMENT

The authors declare no conflicts of interest. We confirm that we have read the Journal's position on issues involved in ethical publication and affirm that this report is consistent with those guidelines.

## Supporting information


Figure S1.
Figure S2.

## Data Availability

The data that support the findings of this study are available on request from the corresponding author. The data are not publicly available due to privacy or ethical restrictions.
