# Supplementary material for: High incidence of Y‐chromosome mosaicism in male and female individuals with mild malformation of cortical development with oligodendroglial hyperplasia in epilepsy
Source: Epilepsia. 2026 May 30;67(9):4907–21. doi: 10.1002/epi.70317 (PMC13592493; doi:10.1002/epi.70317)
Supplement: Supplementary file 1 [file EPI-67-4907-s001.docx]

**Supplementary Material**

**Supplementary Figure 1. DNA methylation–derived copy number variation (CNV) plots for all 29 MOGHE patients.**

A. **CNV plots from Group 1: *SLC35A2* (+); Y chromosome gain (-)**

ID 1. Male

ID 2. Male ID 3. Male ID 4. Female ID 5. Female

ID 6. Female ID 7. Female

ID 8. Female

B. **CNV plots from Group 2: *SLC35A2* (+); Y chromosome gain (+)**

ID 9. Female

ID 10. Female ID 11. Female

ID 12. Female

ID 13. Male

ID 14. Male

ID 15. Male ID 16. Male

ID 17. Male

ID 18. Male

ID 19. Male

ID 20. Male

A. **CNV plots from Group 3: *SLC35A2* (-); Y chromosome gain (+)**

ID 21. Female

ID 22. Male

ID 23. Male

ID 24. Male

ID 25. Male

ID 26. Male ID 27. Male

ID 28. Male

ID 29. Male

Genome-wide CNV profiles are shown for each individual, spanning chromosomes 1 through 22, X, and Y. Log₂ copy number ratios are plotted on the y-axis. Patients are grouped following the genetically defined subgroups described in the manuscript: Group 1 (SLC+/Y–), Group 2 (SLC+/Y+), and Group 3 (SLC–/Y+). These plots provide a complete overview of CNV patterns in the cohort.

**Supplementary Figure 2. FISH Analysis of Y Chromosome structural integrity in MOGHE with Y Chromosomal Gain**

**Legend to Supplementary Figure 2**: FISH analysis of Y chromosome configuration in FFPE brain tissue from patients with MOGHE and Y chromosomal gain. FISH was performed on FFPE brain tissue sections from three patients diagnosed with MOGHE and Y chromosomal gain: Patient ID 9 (female, first row), Patient ID 17 (male, second row), and Patient ID 28 (male, third row). Three probes were used: (i) a probe targeting the centromere of chromosome X (CENX; green), (ii) a probe targeting the centromere of chromosome Y (CENY; red), and (iii) a probe targeting sequences mapping to Yq12 specific for the classical satellite III centromeric region DYZ1 of chromosome Y (Yq12; green). Column 1 (a, e, i): merged RGB images from all channels. Column 2 (b, f, l): merged green and red channels. Green signals (white circles) indicate CENX; red signals (white squares) indicate CENY. The Yq12 probe signal overlapped with CENY in rows 1 and 2. In the female case (row 1), and in the male case (row 2) the Yq12 signal colocalized with CENY and is masked by the centromeric Y signal, indicating that the Y chromosome was intact. In patient ID28 (row 3), however, we observed a partial spatial separation and a smeared appearance of the Yq12 signal, which allowed its distinction from the CENY signal. Column 3 (c, g, m): green channel only, highlighting CENX signals (white circles) and Yq12 signals (white squares). In the female case (row 1), two CENX signals were visible, consistent with a XX karyotype. Column 4 (d, h, n): red channel only, showing CENY signals.
